# Urgent endovascular mycotic aortic arch aneurysm repair using in situ laser fenestration and selective arterial perfusion with venoarterial extracorporeal membrane oxygenation

**DOI:** 10.1016/j.jvscit.2023.101345

**Published:** 2023-10-10

**Authors:** Maysam Shehab, Kevin Mani, Marek Kuzniar, Shinji Miyamoto, Sten Lindgren, Anders Wanhainen

**Affiliations:** a Department of Surgical Sciences, Vascular Surgery, Uppsala University, Uppsala, Sweden; b Department of Cardiovascular Surgery, Oita University, Oita, Japan; c Department of Surgical and Perioperative Sciences, Umeå University, Umeå, Sweden

**Keywords:** Aortic arch, FTEVAR, In situ laser fenestration, Mycotic, Stroke, VA-ECMO

## Abstract

In recent years, mycotic aortic aneurysms have been increasingly treated successfully by endovascular means. The introduction of custom-made fenestrated and branched devices, parallel graft techniques, and in situ fenestration has enabled total endovascular treatment also for arch pathologies. We describe a total endovascular repair of a mycotic arch aneurysm with in situ laser fenestration using venoarterial extracorporeal membrane oxygenation to preserve flow to vital organs.

A mycotic aortic aneurysm (MAA) is a rare, but serious, disease, with mortality rates of ≤50% after radical surgical repair. In recent years, abdominal and thoracic MAAs have been increasingly treated successfully by endovascular means.^1^^,^^2^ Recently, the introduction of custom-made fenestrated and branched devices, parallel graft techniques, and in situ fenestration has enabled total endovascular treatment of arch pathologies.^3^, ^4^, ^5^, ^6^

We describe a total endovascular repair of a mycotic arch aneurysm with in situ laser fenestration (ISLF) using venoarterial extracorporeal membrane oxygenation (VA-ECMO) to preserve flow to vital organs.

## Case report

A 75-year-old man presented with high fever and ongoing chest pain. His medical history included previous coronary artery bypass grafting with left internal mammary artery (LIMA) to left anterior descending artery (LAD) bypass, minor stroke without sequelae, atrial fibrillation requiring apixaban (Eliquis; Bristol-Myers Squibb), hypertension, obesity, excessive alcohol consumption, and ongoing long-term treatment with corticosteroids for polymyalgia rheumatica.

**Fig 1:**
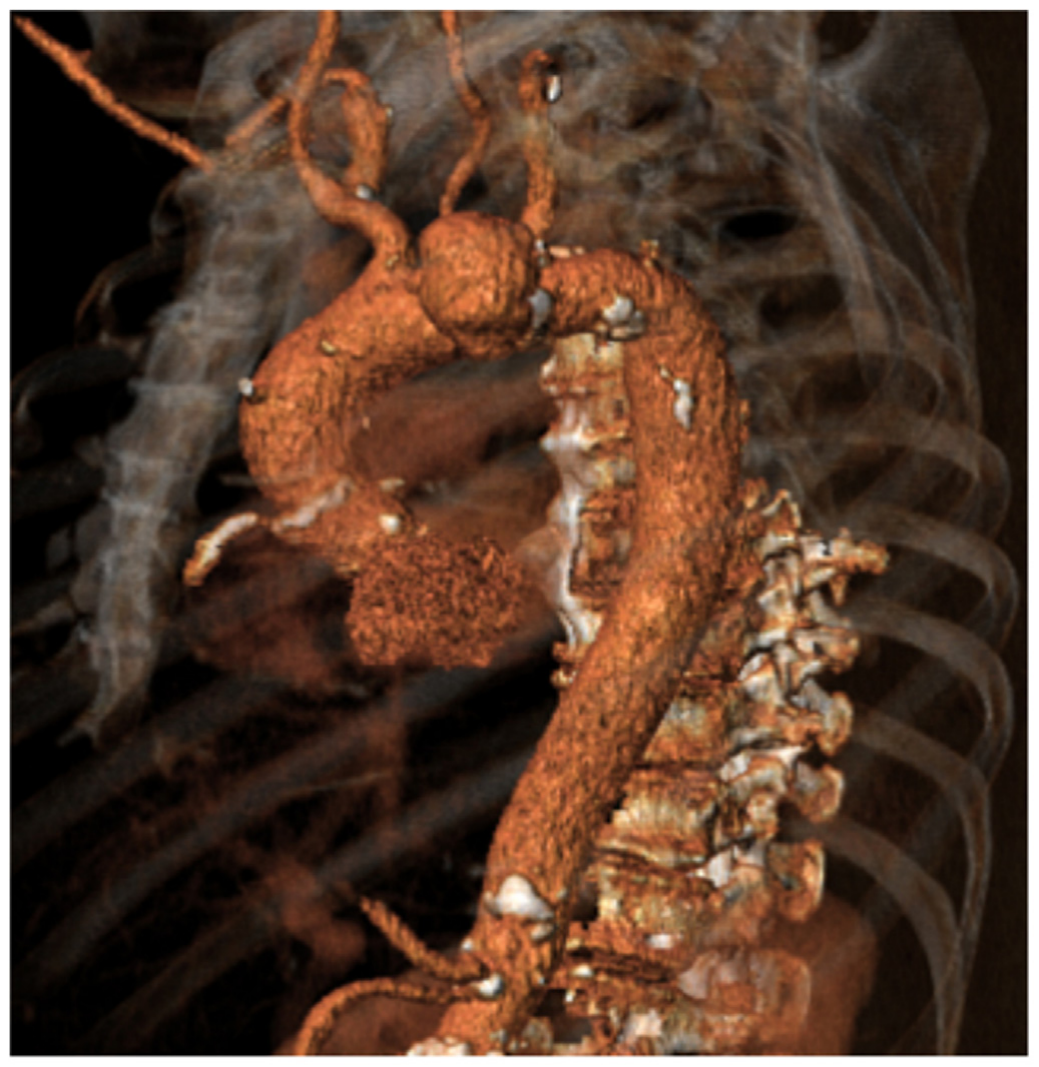
Three-dimensional reconstruction demonstrating a saccular aortic arch aneurysm with 50-mm maximal diameter.

On admission, his vital parameters were normal, his leukocyte count was 13.6 10^9^/L, and his C-reactive protein level was 187 mg/L. Computed tomography (CT) angiography demonstrated a 5-cm saccular aortic arch aneurysm, with a zone 0 proximal landing zone (Fig 1).

The diagnosis of MAA was determined by a combination of the clinical presentation, laboratory test results, and CT findings, as suggested by recent guidelines.^1^ Blood cultures were obtained, and adjuvant wide-spectrum antibiotics were initiated. Echocardiography showed normal left ventricular function and no vegetation, and a coronary angiogram demonstrated a patent LIMA to LAD bypass.

Open surgical arch reconstruction was ruled out due to his comorbidities, operative risk, and ongoing infection. Total endovascular aortic arch repair with a branched arch stent graft was not deemed suitable due to the small diameter of the ascending aorta and arch, compromising an adequate opening of the branches. Therefore, a decision was taken to perform total aortic arch coverage with thoracic endovascular aneurysm repair, followed by ISLF for the supra-aortic vessels, with selective perfusion of the brain and LIMA to LAD bypass with VA-ECMO.

**Fig 2:**
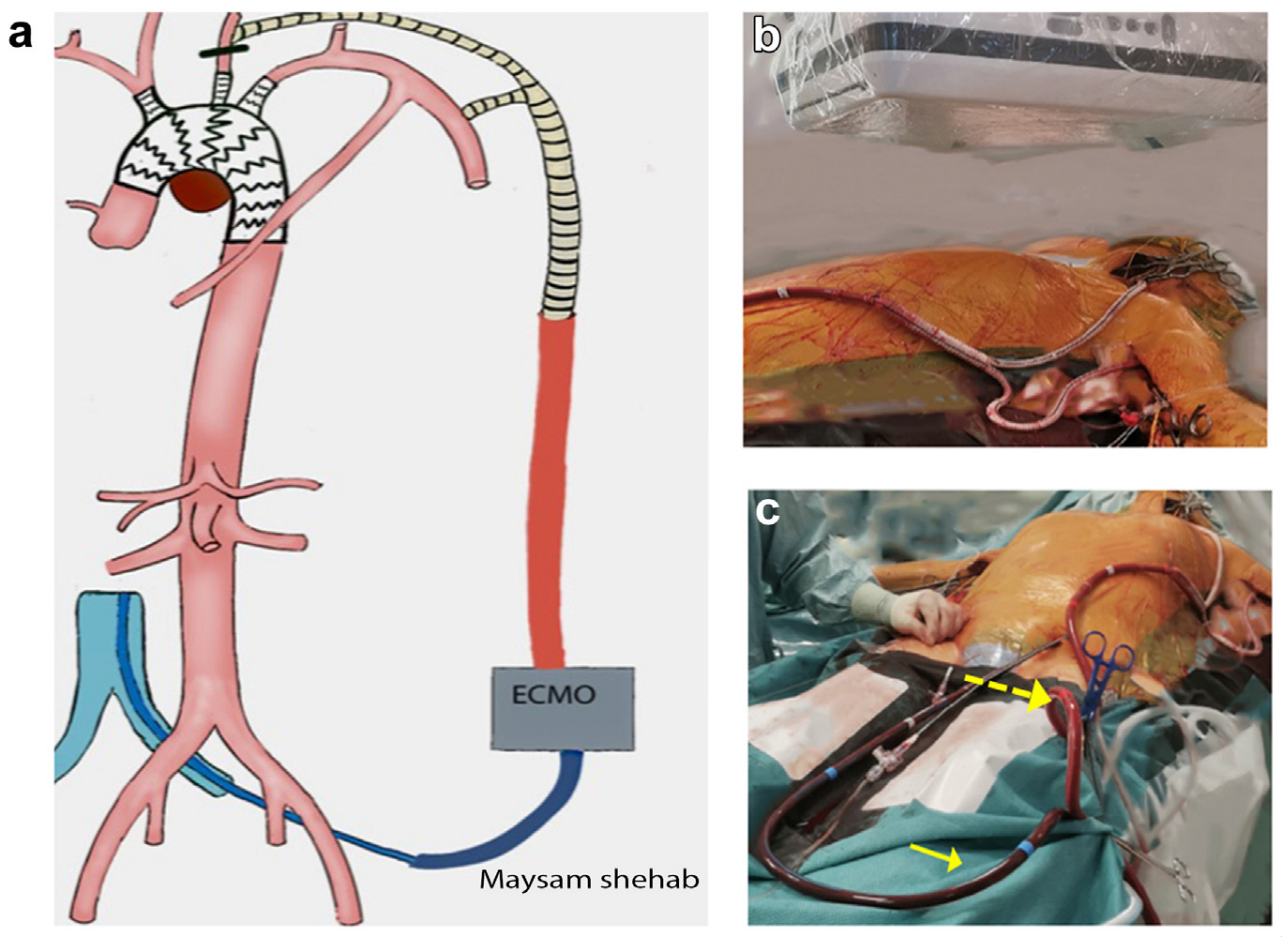
**a,** Illustration of the operative setup, with selective perfusion to the brain and left internal mammary artery (LIMA) bypass, using venoarterial extracorporeal membrane oxygenation (VA-ECMO) via a temporary bifurcated graft with end-to-side anastomosis to the left axillary artery and left common carotid artery (CCA). Proximal clamping of the left CCA was performed to prevent distal embolization, followed by thoracic endovascular aneurysm repair of the arch and in situ laser fenestration (ISLF) of the supra-aortic vessels. **b,** Bifurcated graft with end-to-side anastomosis to the left axillary artery and left CCA. The main body of the graft was connected to the arterial VA-ECMO cannula. **c,** Venous VA-ECMO cannula in the left common femoral vein (*full arrow*) and arterial VA-ECMO cannula with oxygenated blood perfusing the left axillary artery and left carotid artery (*dashed arrow*).

The procedure was performed under general anesthesia with intraoperative neuromonitoring using the INVOS system (Medtronic) in a hybrid operating room. A VA-ECMO system (Getinge Cardiohelp) with attached heart and lung support set was used to maintain cerebral and LIMA perfusion. The left common carotid artery (CCA) and left axillary artery were exposed, and the limbs of a 16 × 8-mm bifurcated Dacron graft were anastomosed end-to-side to the arteries. The arterial VA-ECMO outflow 3/8-in. tube connector was connected to the graft's main body. After ultrasound-guided percutaneous access to the common femoral vein, the ECMO's venous cannula (Medtronic Biomedicus; 21F) was inserted and placed in the right atrium (Fig 2). The left CCA was clamped proximal to the ECMO–bypass anastomosis to prevent cerebral embolism throughout the procedure. The oxygenated and filtered blood was returned to the brain and LIMA through the VA-ECMO arterial cannula. The VA-ECMO initiated a circuit flow of 800 mL/min and maintained a mean arterial pressure of 70 mm Hg. Heparin was administered to maintain an activated clotting time (ACT) >350 seconds. Satisfactory INVOS neuromonitoring was observed. The right brachial artery was exposed, and an 8.5F, 55-cm TourGuide Steerable Sheath (Medtronic Vascular) was inserted and positioned into the brachiocephalic trunk. After ultrasound-guided percutaneous access to the right common femoral artery, a Lunderquist double curved wire (Cook Medical) was positioned in the sinuses of Valsalva. A tapered thoracic stent graft (Cook Zenith alpha, 38 mm × 34 mm × 167 mm) was deployed from the ascending into the descending artery, covering the entire arch.

A 2.3-mm laser catheter (Turbo Elite; Philips Healthcare) was inserted into the steerable sheath positioned in the brachiocephalic trunk. The tip of the steerable sheath was adjusted to align with the ostium of the trunk. Subsequently, retrograde ISLF was performed, after which a 0.018-in. wire was advanced into the aortic arch, and the fenestration was predilated with a 5 × 40-mm balloon (Armada 18; Abbott Cardiovascular). The 0.018-in. wire was exchanged for a 0.035-in. Rosen wire (Cook Medical), and a 10 × 38-mm covered stent (Advanta Atrium V12; Getinge) was deployed. In a similar fashion, retrograde ISLF was performed in the left CCA, and an 8 × 38-mm covered stent graft (Advanta Atrium V12) was deployed. The artery was flushed and sutured, and the carotid artery clamp was removed. Next, the temporary carotid artery shunt was clamped, resulting in a decreased flow rate within the ECMO system to 400 mL/min. Finally, the left brachial artery was punctured, and a third retrograde ISLF was performed for the left subclavian artery, and a 10 × 38-mm covered stent (Advanta Atrium V12) was deployed. The artery was flushed and the arteriotomy was sutured.

**Fig 3:**
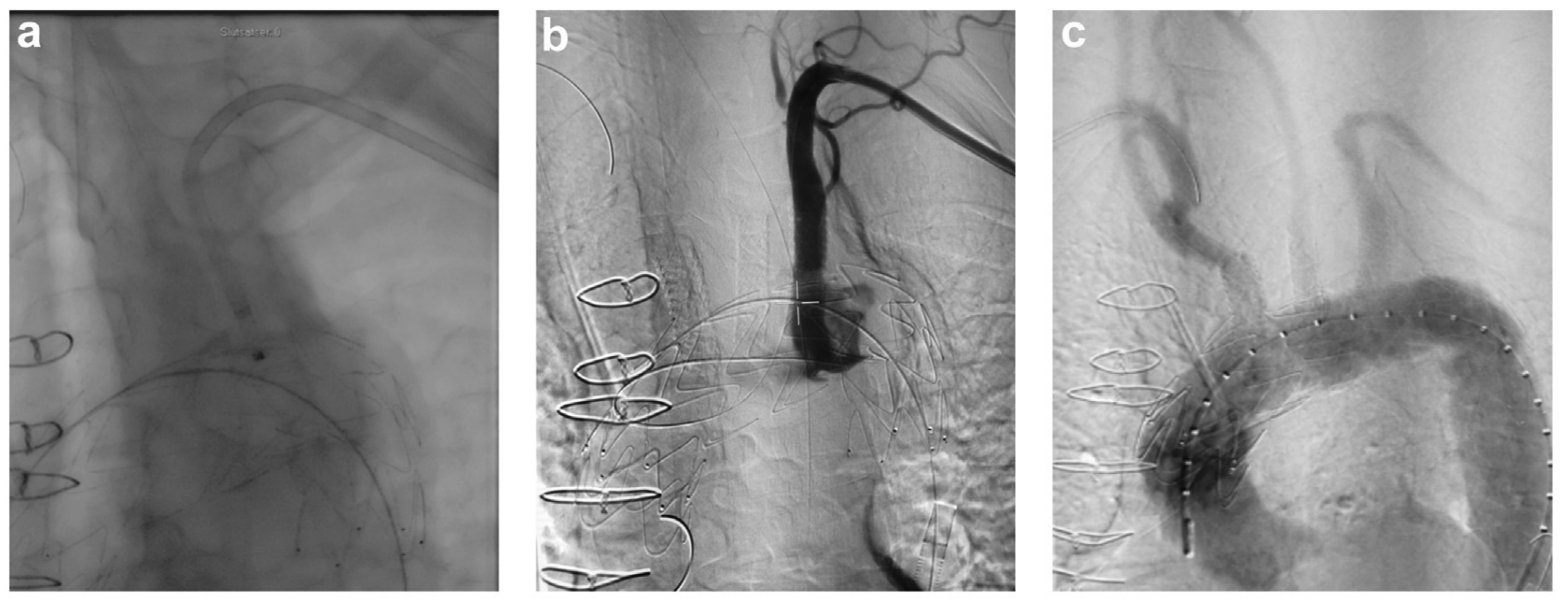
**a,** Retrograde in situ laser fenestration (ISLF) of the aortic stent graft for the left subclavian artery. **b,** Intraoperative angiography of the left subclavian artery bridging stent graft, demonstrating a patent left internal mammary artery (LIMA) to left anterior descending artery (LAD) bypass and vertebral origin. **c,** Completion aortic angiogram showing exclusion of the mycotic arch aneurysm with no major endoleak and good flow of the supra-aortic vessels.

**Fig 4:**
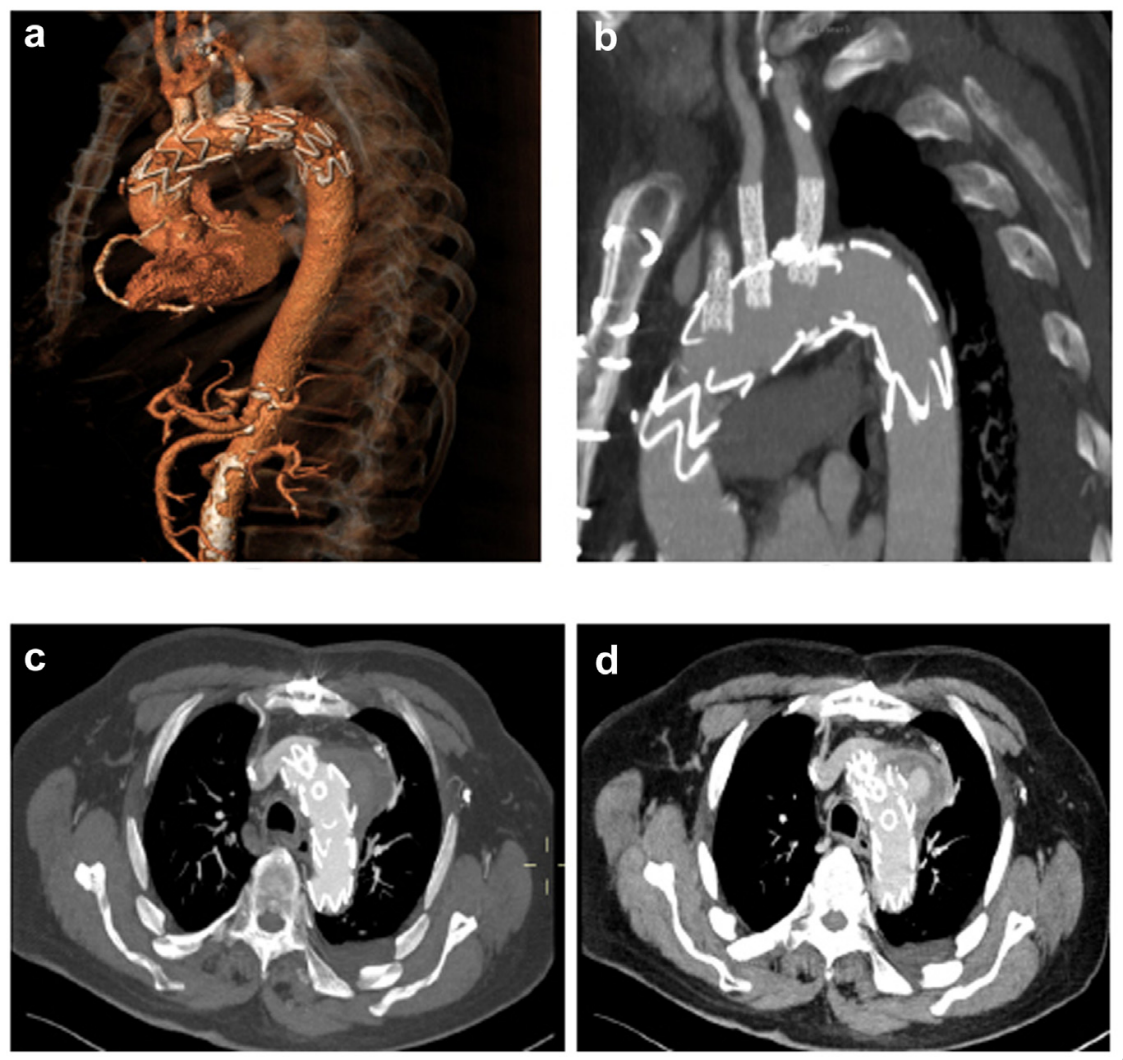
Immediate postoperative computed tomography (CT) angiography (*CTA*) scan. **a,** Three-dimensional reconstruction of the totally endovascularly repaired aortic arch aneurysm. **b,** CTA, sagittal view. **c,** CTA, early arterial phase axial view, with no endoleak present in the aneurysm sac. **d,** CTA, late venous phase axial view, showing endoleak present in the aneurysm sac.

**Fig 5:**
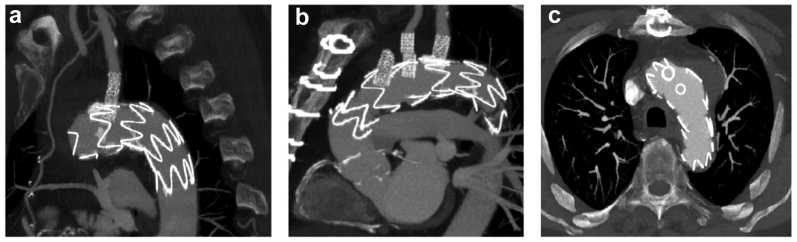
One month follow-up computed tomography scan showing a patent left internal mammary artery (LIMA) to left anterior descending artery (LAD) coronary bypass (*arrow*; **a**), a patent right coronary artery (**b**), and the excluded aneurysmal sac with no remaining visible endoleak (**c**).

The ECMO circuit was weaned off, and the heparin effect was reversed. Subsequently, the bifurcated graft limbs were removed, and the arteries were primarily repaired. A final completion angiogram showed no endoleaks or vessel compromise (Fig 3). The total operating time was 4.5 hours, with 60 minutes of ECMO time. The patient was extubated on the same day, and his postoperative course was uneventful. Postoperative CT angiography demonstrated a satisfactory result and a late phase type II endoleak (Fig 4). The patient was discharged on postoperative day 14 with a continued intravenous antibiotic for ≥6 months. The 1- and 3-month follow up CT scans depicted a thrombosed aneurysm and patent arch bridging stents and LIMA bypass. No retrograde dissection was seen (Fig 5).

The patient provided written informed consent for the report of his case details and imaging studies.

## Discussion

The advancement of new endovascular techniques to treat aortic arch aneurysms in recent years, with specific delivery systems and preloaded fenestrations and inner branches, has improved results to a level that endovascular arch repair has become a viable option for patients with increased risks for open repair. Total endovascular aortic arch repair is a very promising reality, even for emergent arch pathologies that cannot wait for a custom-made solution. For these cases, implantation of an off-the-shelf or in situ fenestrated stent graft should be considered.

A recent systematic review of ISLF for the endovascular management of aortic arch pathologies showed a low stroke rate of 5%.^7^ However, reports of ISLF so far consist of small single-center experiences, resulting in the possibility of selection and publication bias.

A potential limitation with the ISLF method in general, and as an energy-based technology, is the risk of fraying the fabrics during balloon dilation. Experimental studies suggest that the use of multifilament polyethylene terephthalate, followed by dilation with noncompliant balloons ≤8 mm, is the most durable in vitro technique for ISLF.^8^ High-pressure balloons, ≤15 atm, might be required to fully open the bridging stent graft in the ISLF, and the use of flaring balloons could increase the risk of fabric tears.^9^

The ECMO machine provides a temporary mechanical cardiopulmonary support system that has been used to maintain perfusion in critically ill patients. The typical adult ECMO circuit provides between 1 and 6 L/min of blood flow, which can be lower in pediatric ECMO machines, and have heparin-bonded tubing, which allows for lower levels of anticoagulation (ACT, 180-220 seconds). For our patient, due to the low flow in the ECMO circuit, the ACT goal was increased to 300 to 350 seconds to avoid thromboembolic complications in the oxygenator.

## Conclusions

Total ISLF aortic arch repair with selective perfusion and embolic protection via VA-ECMO could provide an alternative for patients who are poor surgical candidates and cannot wait for a customized device. Additional data with longer follow-up are needed to better assess the method's safety and durability.

## Disclosures

None.
